# Autism service barriers for Latine children and their families: a participatory approach to adapting autism diagnostic care

**DOI:** 10.3389/fpsyt.2026.1834721

**Published:** 2026-07-09

**Authors:** Ann Marie Martin, Gisela Perez, Elizabeth Frances Battle, Aaliyah Saunders, Stephanie Pozuelos, Mary Ciccarelli, Angela Paxton, Carrie Leathers, Rebecca McNally Keehn

**Affiliations:** 1 Indiana University School of Medicine, Indianapolis, IN, United States; 2 Indiana University Paul H O’Neill School of Public and Environmental Affairs, Indianapolis, IN, United States; 3 Purdue University, West Lafayette, IN, United States; 4 University of Cincinnati, Cincinnati, OH, United States

**Keywords:** autism, cultural differences, Latino/a/x, limited english proficency, mixed methods research methodology, participatory research

## Abstract

**Introduction:**

Latine children from families with limited English proficiency (LEP) experience barriers to timely autism diagnosis resulting in persistent health inequities. This participatory mixed-methods study examined these barriers and identified multi-level strategies to adapt diagnostic care models for Spanish-speaking Latine families.

**Methods:**

Participants included twelve caregivers of thirteen autistic children with parent reported diagnosis of autism, twelve clinicians, and six care coordinators. All participants completed surveys and participated in qualitative interviews or focus groups.

**Results:**

Quantitative findings revealed significant lag between caregiver’s first developmental concern and diagnosis, limited autism knowledge, and difficulty navigating care. Qualitative results highlighted systemic barriers, including clinician-patient language discordance, interpreter inaccuracies and quality variability, cultural stigma, and long waitlists. Clinicians reported challenges with communicating about autism with Spanish-speaking families and emphasized language and cultural differences as primary barriers. Caregivers described social isolation, mistrust of health systems, and emotional distress compounded by immigration-related stressors.

**Discussion:**

Findings underscore the need for structural adaptation of care approaches beyond translation services.

## Introduction

Autism, a heterogeneous neurodevelopmental condition occurring in 1 in 31 United States (US) children (1), emerges in the early developmental period and is characterized by persistent challenges in social and communication skills and the presence of restricted and repetitive behaviors (2). Historically, children from minoritized backgrounds have faced significant disparities in access to timely autism diagnosis and related care (3). More recently, research suggests that this gap may be closing, with reported autism prevalence rates being higher in racial and ethnic minority children as compared to White children (1). However, substantial disparities in autism prevalence persist for Latine children in some regions of the US (1). Latine children and children from minoritized backgrounds continue to be diagnosed later and at lower rates in certain regions, demonstrate greater developmental impairment, autism symptom severity, and behavioral comorbidities (4, 5) and have poorer access to autism services and resources (6) as compared to non-Latine children. Language and cultural barriers (e.g., stigma, lack of culturally competent care) result in greater unmet needs (7). Specifically, research has suggested racial inequities are a symptom of structural and systemic racism within health care (8), creating a cycle where autistic children are approximately four times more likely to have unmet health needs than children without autism (9). This disparity is a public health concern as later diagnosis and entry into behavioral interventions worsens functional developmental outcomes (10) and increases care costs (11). As such, racial and ethnic autism health inequities result in a large economic cost to society (12). Thus, there is a critical need for improved understanding of the barriers that delay access to autism diagnostic care so that equitable models of early autism diagnosis and detection for Spanish-speaking Latine children can be developed and implemented.

Several drivers of autism diagnostic disparities in Latine communities have been proposed. These include clinician implicit biases towards monolingual Spanish-speaking Latine families (13), structural barriers such as the high cost of services, lack of health insurance, and limited transportation access (14, 15), and cultural factors, including limited autism knowledge (16, 17) and distinct beliefs about child development and milestone acquisition (18). Additionally, immigration-related challenges such as residency status in the United States and citizenship documentation challenges, limited English proficiency (LEP), and low English literacy may further discourage care-seeking (19, 20). LEP is a particularly impactful barrier, contributing to communication breakdowns during clinical encounters, difficulties navigating healthcare and autism service systems, and reduced awareness of autism, which hinders engagement with clinicians (21).

Barriers to care, resulting from the complex interaction between structural, family, and individual factors, are dynamic and may change over time based on sociodemographic factors such as political climates, region of residency (i.e., urbanicity of the area), and level of acculturation. As such, engaging LEP Latine families, a historically underrepresented population in research, is essential to understanding their lived experiences and co-developing and testing autism care models. To date, there are no adapted models of autism diagnostic care for Latine LEP families in the United States, which is a significant problem as Latine children represent the largest and fastest growing racial and ethnic minority group among children in the United States today (22). Most adaptation efforts have occurred in non-English-speaking countries, where common limitations include reliance on translation alone, lack of transparency in adaptation processes, and insufficient attention toward cultural values and customs (23). Additionally, when working with underrepresented communities it is important to avoid imposing Western norms and ignoring contextual factors, thus a mixed methods approach was utilized due to its utility in culturally driven research and ability to ask complex questions framed in the context of cultural factors (24). Thus, the aim of this study was to (1) use a mixed-methods participatory approach to characterize barriers in acquiring an autism diagnosis and related services for children from LEP Spanish-speaking Latine families, and (2) to inform the development of recommendations for adapting models of care to reduce barriers and meet cultural needs.

## Methods

### Participants & recruitment

Participants included 12 Spanish-speaking Latine mothers (*M* = 41.00 years; *SD* = 4.45; 67% LEP) of 13 children (*M* = 8.91 years; *SD* = 4.00; 77% male) with a formal medical or parent-reported diagnosis of autism, 12 clinicians (92% female; 82% White; *M* = 11.42 years in practice; *SD* = 6.91; 41% pediatrics discipline; 83% MD/DO [doctor of medicine]/[doctor of osteopathic medicine]; 58% Good/Excellent Spanish fluency) who provide care for Spanish-speaking Latine populations, and 6 care coordinators (100% female; 50% Latine; *M* = 10.67 years in practice; *SD* = 8.41; 67% social work discipline; 67% Good/Excellent Spanish fluency). Spanish-speaking Latine mothers were recruited from community organizations across the state of Indiana. Eligible clinicians and care coordinators included any provider who had previously provided health care services or care navigation to Spanish-speaking Latine families of children with or at increased likelihood for autism. Clinicians and care coordinators were recruited from several local health care service systems. See **Table 1**.

**Table 1:** Participant demographics.

Demographics	*N*=12
Caregiver Sex (Female)	12 (100)
Caregiver Age (SD)	41.00 (4.45)
Caregiver Race/Ethnicity (Latine)	12 (100)
Caregiver Marital Status (Married)	9 (75)
Caregiver Outside Employment	2 (17)
Income	
<$25,000	1 (14)
$25,000-49,999	5 (71)
$50,000-74,999	1 (14)
Caregiver Years in School (SD)	12.18 (4.00)
Years in United States	17 (9)
Caregiver English Proficiency	
Very Good	2 (17)
Good	2 (17)
Not Good	5 (42)
Does not Speak English	3 (25)
Autistic Child Sex (Male)	10 (77)
Autistic Child Age (SD)	8.91 (3.97)
Perceived Child Autism Severity	
Mild	4 (33)
Moderate	7 (58)
Severe	1 (8)
Insurance Type	
Private	3 (23)
Public	11 (85)

	*N*=12
Clinician Sex (Female)	11 (92)
Clinician Race/Ethnicity	
Asian	1 (9)
Latine	1 (9)
White	10 (82)
Years in Practice M(SD)	11.42 (6.91)
Spanish Proficiency (Fluent)	7 (58%)
Discipline	
Pediatrics	5 (41)
Primary Care	2 (17)
Developmental Behavioral Pediatrics	2 (17)
Pediatric Psychology/Psychiatry	2 (17)
Speech Pathologist	1 (8)

	*N*=6
Care Coordinator Sex (Female)	6 (100)
Care Coordinator Race/Ethnicity	
Latine	3 (50)
White	3 (50)
Years in Practice M (SD)	10.67 (8.41)
Spanish Proficiency (Fluent)	
Discipline	
Social Work	4 (67)
Bilingual Resource Specialist	2 (33)

All data presented as n (%) unless otherwise noted. Percentages are calculated based on those children for which data are available.

### Measures

#### Clinician and care coordinator survey

Clinicians and care coordinators completed a Likert-scale survey in English with 25 questions across 4 domains (knowledge about diagnosis and treatment of autism (clinician survey only), barriers to autism assessment and diagnosis, language interpretation and services, and demographics). The survey was adapted from Zuckerman et al. (13) with 4 additional demographic questions added specific to regional health care models.

#### Caregiver survey

Spanish-speaking Latine caregivers completed the Autism Healthcare Knowledge and Satisfaction Survey (18) in Spanish. The survey consists of 86 items across 8 domains (child’s autism, the autism diagnosis process, child’s current therapy services, parent beliefs about autism, community views and knowledge about autism, changing health care for autism, and parent and child demographics); items are rated on a 4-point Likert scale from “strongly disagree” to “strongly agree.”

### Procedures

This study was approved by the Indiana University Institutional Review Board (15008). *Survey Administration.* Participants contacted study personnel via phone after viewing study flyers. Participants were provided more information by phone regarding study procedures. Those who agreed to participate were emailed the study information sheet and a link via Redcap to complete the survey and were scheduled for an interview or focus group. After surveys were completed, a research assistant reviewed the survey for completeness and collected any missing data at the time of the interview or during a follow-up call.

### Interviews and focus groups

Bilingual Latine researchers (AMM, GP) facilitated all caregiver focus group discussions (three total; Group 1 *n* = 2; Group 2 *n* = 3; Group 3 *n* = 4) and caregiver, clinician, and care coordinator interviews (20 total) via Zoom Health. Interviews were scheduled for 60 minutes, and focus groups were scheduled for 90 minutes. Participants were provided a gift card for participation. The participant list for each focus group was based on participant availability. Each focus group was overbooked at the time of scheduling to compensate for potential attrition.

Each session began with an overview of the study information sheet, which was read aloud by the moderator and included an explanation of the study purpose, ethical guidelines, and notification of audio recording. For focus groups, ground rules were then reviewed: confidentiality, respect and validation of the experiences and narratives of all members in the group, and anyone who wishes to speak should have the opportunity. The clinician and care coordinator interviews were conducted in English, while the caregiver focus groups were conducted in Spanish.

### Development of moderator’s guide

The lead bilingual researcher (AMM) developed the moderator’s guide based on prior clinical experience, review of the literature, and consultation with experts in the field. Specifically, content validity of the guide was established by a literature review of autism services adapted for Latine populations and by consensus of the research team and consulted field experts. The guide was reviewed and approved by all research team members. The clinician and care coordinator interview questions were centered around 2 domains: (1) clinical experiences working with Latine populations, and (2) clinical experiences serving Latine families with children with developmental delays. Spanish focus group questions were centered around 4 domains: (1) prior to autism diagnosis, (2) navigating the diagnostic evaluation, (3) post-diagnosis and service navigation, and (4) other cultural factors and caregiving experiences. Interviews followed a semi-structured guide with open-ended, narrative questions designed to elicit rich stories of autism diagnostic experiences, barriers and facilitators to care, and experiences related to cultural factors. Follow up questions were asked when necessary to clarify narratives, and conversations were continued until saturation of themes was achieved. The moderator’s guide was used for both the focus groups and interviews.

All sessions were audio recorded and transcribed verbatim in their original language by the bilingual research team. One member of the research team observed each focus group and took field notes documenting mood, tone, facial expressions, and emotional expressions that were observed in the discussions. Social enactment cues were included in the transcript when important to the context of the interaction. Debriefing sessions took place immediately after the focus groups and recurrent meetings were held to review the transcribed recordings for accuracy.

## Statistical analyses

An explanatory sequential research design (25) was used in which quantitative and qualitative data collection occurred independently and quantitative data guided the qualitative strand (25).

### Quantitative analyses

Version 31 of IBM SPSS was used for statistical analysis. Descriptive analyses were calculated. A series of chi-square and t-tests were performed to determine whether survey responses (i.e., caregiver beliefs about autism, community views and knowledge about autism, and selected items regarding the autism diagnostic process) varied by demographic variables (i.e., child age, child sex, and caregiver perceived level of severity). Distributional assumptions were evaluated using visual inspection and indices of skewness and kurtosis. Although Likert scale data are ordinal, prior research supports their treatment as continuous under certain conditions (26). Given the relatively small sample size, sensitivity analyses using non-parametric tests (i.e., Mann-Whitney U and Kruskal-Wallis) were conducted to assess robustness of findings and results were consistent across analytic approaches, therefore only parametric results are presented for brevity.

### Qualitative analyses

The present study used thematic content analysis (27) and conversation analysis (28) for an applied analytic approach often used in health services research for disadvantaged or marginalized groups with specific cultural needs (see 29). A list of preliminary themes was created from a review of the literature and informed by the quantitative results and served as a theoretically informed framework and deductive approach to developing qualitative themes. Research team members coded independently and then met weekly and discussed codes until consensus was reached on final themes and subthemes in an iterative and reflexive process (30). Special attention was placed on high-frequency themes as well as lower-frequency themes paired with a strong emotional response (e.g., crying) by participants. Inter-rater reliability was calculated and was consistently above 80 percent (Interviews=73%; FG1 = 82%; FG2 = 91%; FG3 = 92%) after the initial interview coding meetings when codes were still being generated and adapted, indicating acceptable inter-rater reliability. Version 15 of Lumivero NVivo was used. Saturation and richness were determined when there was a lack of novel patterns and codes identified in new transcripts.

## Results

### Quantitative results

#### Caregiver quantitative results

See **Table 2** for item-level survey data. On average, caregivers first noted differences in their child at age one and a half years (M = 1.75 years; SD = 1.20) and first discussed their concerns with clinicians when their child was two and a half years old (M = 2.50 years; SD = 1.70). Despite these early signs and conversations, the average age of child diagnosis was over four years (M = 4.20 years; SD = 1.50; range 2–7 years).

**Table 2:** Caregiver survey results.

Item	Mean	Definitely Disagree	Disagree	Agree	Definitely Agree
Before a doctor diagnosed my child with ASD...					
I thought there was a problem with my child’s behavior	3.08	1 (8)	2 (16)	4(33)	5 (42)
My spouse/partner thought there was a problem with my child’s behavior	2.92	1 (8)	4 (33)	2(17)	5(42)
A doctor or nurse said there was a problem with my child’s behavior	3.00	0	4(36)	3(27)	4(36)
Family members/elders thought there was a problem with my child’s behavior	2.83	1 (8)	5 (42)	1(8)	5(42)
The process of getting an ASD diagnosis caused stress within my family	3.08	0	3(25)	5(42)	4(33)
I knew a lot about ASD	1.42	7(58)	5(42)	0	0
Doctors or nurses gave me useful information about ASD	2.27	2(18)	4(36)	5(45)	0
It was difficult to understand how the medical system worked	2.82	2(18)	2(18)	3(27)	4(36)
It was difficult to trust the doctors’ or nurses’ advice	2.45	0(0)	7(63)	3(27)	1(9)
I knew where to go for help	1.83	5(42)	5(42)	1(8)	1(8)
I got all the help I needed coordinating medical care for my child	2.36	3(27)	2(18)	5(46)	1(9)
Appointments were uncomfortable for my child	2.36	1(9)	6(55)	3(27)	1(9)
It seemed like my child got an ASD evaluation quickly	2.33	3(25)	5(42)	1(8)	3(25)
I had to travel a long distance to get an ASD evaluation	2.09	4(36)	4(36)	1(9)	2(18)
Getting an ASD evaluation was expensive for my family	2.27	4(36)	3(27)	1(9)	3(27)

	Mean	Definitely Disagree	Disagree	Agree	Definitely Agree	Does Not Apply
Medical Interpreters were available when we needed them	3.36	1(9)	2(18)	3(27)	2(18)	3(27)
Medical interpreters helped me understand what doctors and nurses were saying	3.50	1(8)	2(17)	3(25)	2(17)	4(33)
Doctors thought my child’s problems were caused because we spoke Spanish at home	2.17	4(33)	4(33)	2(17)	2(17)	0
I was afraid to ask for help because of legal issues in our family	3.08	4(33)	1(8)	1(8)	2(17)	4(33)

	Mean	Definitely Disagree	Disagree	Agree	Definitely Agree
Parent Beliefs about ASD					
My child’s ASD is likely to be lifelong rather than temporary	3.20	1(10)	0	5(50)	4(40)
The challenges related to my child’s ASD can be decreased with treatment	3.20	0	1(10)	6(60)	3(30)
I have the power to change my child’s ASD	2.20	3(30)	4(40)	1(10)	2(20)
My child’s ASD is a mystery to me	2.20	2(22)	4(44)	2(22)	1(11)
When I think about my child’s ASD, I get upset	2.18	3(27)	4(36)	3(27)	1(9)
My child’s ASD has major consequences on his/her life	2.67	0	6(50)	4(33)	2(17)
It was God’s plan for my child to have ASD	3.36	0	2(18)	3(27)	6(55)
Community Views and Knowledge about ASDs					
People in my community...					
Tell me that my child with ASD will “grow out of it”	2.08	4(33)	4(33)	3(25)	1(8)
Think that ASD is a result of bad parenting or lack of discipline	1.92	6(50)	2(17)	3(25)	1(8)
Think that ASD only happens in the United States	1.67	7(58)	2(17)	3(25)	0
Want to learn about ASD	2.55	3(27)	1(9)	5(46)	2(18)
Think that ASD is something to be ashamed of	2.09	4(36)	3(27)	3(27)	1(9)
Think that ASD is a medical condition	2.92	1(8)	1(8)	8(67)	2(17)
Have little information or knowledge about ASD	3.18	1(9)	2(18)	2(18)	6(55)
Are uncomfortable around my child with ASD	2.27	3(27)	4(36)	2(18)	2(18)
Try to help my child and our family	2.73	1(9)	1(9)	9(82)	0
Say children with ASD are “stupid,” “crazy,” or other hurtful words	1.82	6(55)	1(9)	4(36)	0
Think children with ASD have special abilities	3.30	0	1(10)	5(50)	4(40)

All data presented as n (%) unless otherwise noted. Percentages are calculated based on those children for which data are available. Means are calculated for individual Likert scale questions, higher scores indicate stronger agreement with each statement, viewpoint, or experience.

#### The autism diagnosis process

Regarding early behavior problems before the diagnosis, a majority of caregivers reported that they thought there was a problem with their child’s behavior (*n* = 9; 75%), over half of their spouses thought there was a problem with their child’s behavior (*n* = 7; 59%), and about half of caregivers reported that their family members thought there was a problem with their child’s behavior (*n* = 6; 50%). One hundred percent of caregivers reported limited autism knowledge (*n* = 12). Less than half of caregivers reported receiving useful information about autism from health care clinicians at the time of diagnosis (*n* = 5; 45%). Sixty three percent of caregivers reported that it was difficult to understand how the medical system worked (*n* = 7) and a majority of caregivers did not know where to go for help (*n* = 10; 84%). Most caregivers were able to get an evaluation locally (*n* = 8; 72%) and the evaluation was covered by insurance for 63% of families. Thirty-four percent of caregivers (*n* = 4) reported that a doctor told them their child’s delay was caused by speaking Spanish at home and 25% of caregivers (n=3) hesitated on pursuing services due to documentation or legal issues within the family.

#### Parent beliefs and community views about autism

Nearly all caregivers (*n* = 9; 90%) agreed that their child’s autism diagnosis was likely to be lifelong (rather than temporary) and that challenges could be reduced through accessing treatment (*n* = 9; 90%). Over half of caregivers felt that they did not have the power to change their child’s autism (*n* = 7; 70%). Thirty-six percent of caregivers (*n* = 4) endorsed feeling upset when they think about their child’s autism. Caregivers were split on whether their child’s autism has major consequences on his/her life, with 50% of caregivers agreeing with the statement, and 50% disagreeing (*n* = 6). All but two caregivers endorsed that it was God’s plan for their child to have autism (*n* = 9; 82%). Regarding community views, most caregivers endorsed that community members do not tell them that their child will grow out of their autism diagnosis (*n* = 8; 66%), do not think autism is exclusively a disorder that occurs in the United States (n=9; 75%), and do want to learn more about autism (*n* = 7; 64%). However, 36% (*n* = 4) endorsed that community members are uncomfortable around their autistic child and say autistic children are “crazy” or other hurtful language, and 90% (*n* = 9) reported that community members think autistic children have special powers.

#### Changing health care for autism

When queried about the best ways to help families get more information about autism, 75% (*n* = 9) endorsed doctors and nurses should tell parents more about all the steps to get an autism diagnosis. Fifty-eight percent (*n* = 7) endorsed that television, radio and newspapers should have more stories about autism, 42% (*n* = 5) endorsed that medical offices should have more information for parents about autism, and 33% (*n* = 4) endorsed that doctors and nurses should be more knowledgeable about autism. When queried about the best ways to improve the process of getting an autism diagnosis, 83% (n=10) endorsed that doctors and nurses should act faster when parents have concerns about their child’s development. Sixty-seven percent (*n* = 8) endorsed that interpreters should be more available for families that need them, 33% (*n* = 4) endorsed that doctors and nurses should give out checklists for autism signs during routine child checks, and 33% (*n* = 4) endorsed that clinics should help families make appointments for autism services.

Significant associations were found between child/caregiver demographic variables (i.e., child age, child sex, and caregiver perceived level of severity) and survey questions. For example, there were significant associations between the child’s age and how caregivers responded to: (1) “my child’s autism has major consequences on his/her life”; t= -2.6; *p* = .03; *d* = -1.5), such that caregivers of older children were more likely to endorse negative lifelong consequences; (2) “It was God’s plan for my child to have autism” (t= -2.4; *p* = .04; *d* = 1.9), such that caregivers of younger children were more likely to agree; (3) “people in my community think that autism is a result of bad parents or lack of discipline” (t= -2.3; *p* = .02; *d* = -1.4) such that caregivers of older children were more likely to agree with the statement, (4) “people in my community try to help my child and our family” (t= -2.2; *p* = .05; *d* = -1.7) such that caregivers of younger children were more likely to disagree; (5) “I got all the help I needed coordinating medical care for my child” (t= -3.7; *p* = .005; *d* = -2.2), such that caregivers of younger children were more likely to disagree with that statement; and (6) “it was difficult to trust the doctors’ or nurses’ advice” (t= -2.7; *p* = .03; *d* = -1.7) such that caregivers of older children were more likely to agree with that statement. When analyzing caregiver responses by child sex, there was one significant association. Caregivers of males were more likely to agree with the statement, “people in my community try to help my child and our family” (*X^2^* = 6.5; *p* = .01; φ = -.7). Finally, a significant association was found between caregiver perceptions of their child’s autism severity and how they responded to “the challenges related to my child’s autism can be decreased with treatment” (*X^2^* = 10.0; *p* = .007; φ = 1.0). Specifically, caregivers of children requiring very substantial support were more likely to disagree with the statement. All other association p’s >.10.

### Caregiver qualitative findings

Eight major themes emerged from the focus groups and interviews: (1) limited knowledge about autism prior to the diagnosis; (2) experiences leading up to the evaluation; (3) experiences with the diagnostic evaluation; (4) reaction to the diagnosis; (5) conversations with clinicians; (6) post-diagnostic care; (7) community and supports; and (8) worry about the future. Selected quotes are provided below and in **Table 3**.

**Table 3:** Qualitative themes.

Domain	Theme	Sample Quotes
Caregivers		
Pre-Diagnosis	• Caregivers endorse limited knowledge of autism prior to diagnosis • Experiences leading up to the evaluation	*“And when he was diagnosed at around 2 years, the doctor told me that it was too early to receive a diagnosis...they told me that it was like pre-autism, that it wasn’t 100% but it would be something like autism. But I didn’t even understand what autism was and I thought ‘that won’t happen, he’ll outgrow it’...and so he was diagnosed at 3 years old, my husband didn't accept it”* *“In Mexico, they still think it is a taboo...children who behave badly are crazy. They just are not diagnosed with something, because the mother doesn’t give them a spanking, they do not give them good discipline, and they are pure tantrums”* *“It was very difficult, like completely. Unless maybe I was used to another way of things in [previous city], it’s very different, here there’s only blocks, bunch of barriers, your insurance that you don’t qualify, that this insurance doesn’t cover this. Actually, I still do not understand. My child still doesn’t receive therapies, he just receives one thirty-minute therapy a week, because I don’t understand. I do not understand what is happening with the insurance.”*
Evaluation	• Experiences with the diagnostic evaluation • Reaction to the diagnosis • Conversations with providers	*“But I had an interpreter that several times didn’t say what I wanted to say, they would change my words, or so I was thinking what if he’s not saying the same thing when the doctor is talking to me. No, he didn’t correctly say everything I wanted him to say, I would repeat it again, can you tell him this, this, and this, and they would say yeah I already told him, that’s all what they would say...and because of embarrassment I would stay quiet and wouldn’t say anything, but I continued thinking if in reality it was the same or the opposite. The doctor would say one thing, and he would try to tell me something different. I think that this person lacked a lot despite being from an agency. He lacked understanding the concept of what we were doing to be able to give a good translation”* *“It’s almost as if I don’t have a husband, in that sense. And the rest of our families, I don’t care. They know he has autism, and they don’t help me, and I don’t care...I hope God doesn’t take me soon because I don’t know what my son would do without me. I always tell my eldest son who’s 15, ‘if God takes me, take care of him. Your dad doesn’t know how to help him, he doesn’t know.’”* *“Yes, I think [the clinician] had a hard time adjusting to the culture. So, when he spoke, he spoke straight to the translator. And so, maybe it’s because I didn’t understand him to begin with, but it felt distant”* *“I would say it went as normal, like any doctor, but they’re not comforting. It’s more about do you understand what I’m saying to you? Just with the word autism I had an idea since it’s the same in English, but in speaking, I don’t like how she gave the diagnosis.” “They didn’t express it with kindness.”*
After the Diagnosis	• Post-diagnostic Care • Community and supports • Worry about the future	*“When I got home, I looked through all the documents the doctor gave me to try and figure out what was going on. She gave me resources for ABA institutions. I realized they were centers. I began to ask for help to set up meetings because I don’t speak English...and the wait was very long. I waited almost a year for them to find a spot for my girl because the centers are small and reduced.”* *“The mother of my ex-husband, she told me that [autism] didn’t exist, that he was just a spoiled child, that in Mexico [autism] doesn’t exist. I talked to my family in Mexico, and they said if you give him medication it will be your fault if that child is all drugged up all the time, like a zombie. If you hurt his liver, it will be your fault and instead pray to God, give him discipline...just in the United States only mental illness existed.”* *“Unfortunately, I couldn’t have another [child], but he is my biggest worry. That he knows how to move forward in the future. When I am no longer here, I won’t know what will happen.”*
Clinician/Care-coordinator		
Pre-diagnosis	• Unique patient demographics • Barriers to care and evaluation	*[my patients are] “lower socioeconomic status. Many of them don’t have insurance and we have a safety net program they can use.”* *“However, [the patients] that they’re new, they have a week or less than a month [in the US], those are the one who are coming to the clinic and they’re needing a community health worker right now [because] they’re going through food insecurity.”* *“there’s a lot of fear of calling and not being able to access someone who speaks Spanish, their language, and then you know having to navigate that.”* *[Assessment clinics] “don’t have doctors that speak Spanish. [The families] get hung up on when somebody calls either trying to make an appointment or get information and [the staff] don’t speak Spanish. Most of my families are trying to get autism evaluation and they call locations and [are] told that they can’t help them because they don’t have anyone who speaking Spanish there. So, they get turned away.”*
During the evaluation/after the diagnosis	• Challenges providing care in Spanish • Cultural factors that impact shared understanding	*“I think [interpreters] definitely does put a level of separation between the patient and me.”* *“No, so I am an American I am not Hispanic in any way I did not grow up speaking Spanish. I tried to learn more [Spanish] language and then I’ve just been practicing. So there are things that I want to express that I can’t express [well] but I can say it in a very second or third grade level which is actually probably easy to understand.”* *“I don’t have enough support like my medical assistants don’t speak Spanish, we have been trying to hire medical assistants that speak Spanish, I don’t know if we don’t pay as much as hospitals or what but we haven’t been able to find [one] and then [my organization] prioritizes bilingual workers at other locations. So I don’t really have the support staff to help me with [patient care in Spanish].”* *“I feel that culturally Latino families when I ask them for their input to do shared decision making, they sort of look at me like, ‘Doctora that’s your job, why are you asking me?’ Or they need a little more time and comfort to be able to offer something that won’t come off as an insult to me, so I think that is a cultural barrier to shared decision making for sure.”* *“Either families are hesitant [to accept services] because it’s in the home because there are maybe a bunch of people living in the home, [or] our Spanish-speaking families a lot of times they’ll come and live with extended family. But again Spanish families are very hesitant to let people into their home. I think that’s a very personal thing to do right? To have someone that you don’t know come into your home. SO sometimes there can be hesitancy around that.”* *“It’s a very patriarchal way that they operate. So I try to read that and keep that in mind and make recommendations that are receptive to the family, but also will be helpful for kids and saying it in the way that it will be receptive can be a challenge just cause I don’t always know what they prefer and what I should be saying.”*

Regarding knowledge about autism prior to their child’s diagnosis, only two caregivers had heard of autism and one of them had looked up symptoms to confirm her suspicions about her child’s early challenges. The rest of the caregivers (*n* = 10) had no knowledge about autism. One caregiver reflected on this by saying, “I also did not know about autism, and I only knew about ‘special kids’ that’s what I call them, ‘special kids,’ but I did not understand what that meant until I lived it” (PID#4). Some caregivers reflected on incorrect information about autism they had been exposed to which made the diagnostic process and subsequent emotional processing more challenging. “One can’t understand other families until they understand it themselves. I didn’t know much, I would call them rude, that they would just yell and yell but I didn’t know what they were feeling inside” (PID#4). With regards to experiences leading up to evaluation, several caregivers (*n* = 6) reported long waitlists (i.e., over a year) and challenges with lack of bilingual clinicians or need for interpretation services. One mother in response to being asked about challenges getting a diagnosis said, “Yes, it was rough for me, I had to, not in my city, but go to another center that had translators, so I had to incur costs that I had not planned for” (PID#15). In discussing the evaluation itself, the theme of interpreters came up in several different contexts. Although caregivers were thankful for evaluation centers that offer interpreter services, four mothers specifically described challenges with miscommunication that occurred as a result of poor-quality interpreters, stating “but I had an interpreter that several times didn’t say what I wanted to say, they would change my words,…I would repeat it again, can you tell him this, and they would say yeah I already told him, that’s all they would say” (PID#11). Reactions to the diagnosis were largely split between negative feelings (*n* = 6), sadness (*n* = 4), and denial (*n* = 4), with few caregivers reporting positive reactions (*n* = 1) or an increase in familial and community support (*n* = 1). Most caregivers felt a strain in relationships after their child’s diagnosis, with one mother sharing, “my husband is very reserved, very country [old-fashioned]. It’s been 3 years that he understands a little more. But he doesn’t help … he doesn’t support me. He knows and understands he has autism but he doesn’t help me” (PID#4).

With regards to conversations with heath care clinicians, two mothers reported challenging interactions, specifically describing a lack of empathy. For example, one mother said, “they don’t express [autism] as something positive, something to give us hope. We spoke more at the school, and they gave us a better experience” (PID#15). In addition, five mothers shared that they preferred Latine doctors due to judgmental comments from non-Latine doctors regarding culturally normative expressed affection between parent and child (*n* = 2) and clinician comments that their child’s problems were due to speaking Spanish at home (*n* = 3). Nearly all caregivers endorsed (*n* = 10) challenges navigating and coordinating post-diagnostic care. The most common challenges included difficulty finding information in Spanish (*n* = 3), a lack of professional (e.g. social work) support and caregiver therapeutic access (*n* = 3). Several mothers endorsed a lack of community involvement (*n* = 5) and three mothers reflected on their experience of community stigma, highlighted by one mother’s comment that “suddenly he would go “EEE” like a little squirrel and I would tell him to be quiet because people were beginning to stare … and I would pretend like he wasn’t my son, stuff like that, it’s what you deal with after the diagnosis” (PID#4). Moreover, while a few caregivers received emotional support (*n* = 2) or unsolicited advice from extended family members (*n* = 2), the vast majority of mothers (*n* = 6) only include their spouse and older children in medical and therapeutic decisions for the child with autism. Finally, when queried about the best and hardest parts of raising a child with autism, multiple caregivers (*n* = 4) reported intense worry about the future and what the future holds for their child. Meanwhile positives included their child’s “innocence” (*n* = 5), and one mother described her child stating, “he is a blessing received, or an angel that God sent me, a little angel” (PID#13).

### Clinician/care coordinator quantitative results

See **Table 4** for clinician item level survey results. In regard to knowledge about autism, 67% (*n* = 8) of clinicians rated themselves as being “very” knowledgeable about autism and 33% (*n* = 4) rating themselves as “somewhat” knowledgeable about autism. Clinicians rated other pediatricians slightly lower in autism knowledge (e.g., 83% somewhat; 17% very). Parents of non-Latine White (NLW) and parents of Black children were rated with nearly equivalent levels of autism knowledge (NLW: 50% not very, 42% somewhat, and 8% very knowledgeable; Black: 50% not very, 50% somewhat). English and Spanish speaking parents of Latine children where perceived to have reduced knowledge about autism, with Spanish-speaking parents receiving the lowest ratings on average (English: 8% not at all, 58% not very, 33% somewhat; Spanish: 9% not at all, 73% not very, 18% somewhat). Of note, when queried how difficult it is to recognize the signs and symptoms of autism across the four demographic groups (e.g., NLW, Black, English-speaking Latine, Spanish-speaking Latine), clinicians reported the greatest difficulty recognizing autism in Latine children from Spanish speaking households with 58% of clinicians (*n* = 7) reporting it to be “somewhat” or “very” difficult to recognize.

**Table 4:** Clinician and care coordinator survey results.

Item	Mean (SD)	Not at all	Not Very	Somewhat	Very
How knowledgeable are each of the following about autism?					
Yourself	3.67 (0.50)	0	0	4 (33)	8 (67)
Most pediatricians	3.17 (0.40)	0	0	10 (83)	2 (17)
Parents of non-Latine White children	2.58 (0.70)	0	6 (50)	5 (42)	1 (8)
Parents of Latine children in primarily English-speaking families	2.25 (0.60)	1 (8)	7 (58)	4 (33)	0
Parents of Latine children in primarily Spanish-speaking families	2.09 (0.50)	1 (9)	8 (73)	2 (18)	0
Parents of African-American children	2.50 (0.50)	0	6 (50)	6 (50)	0

Identifying Barriers to Autism Assessment and Diagnosis						
Item	Mean (SD)	Never	Rarely	Sometimes	Often	Frequently
Based on your experience, how frequently do each of the following possible barriers affect autism diagnosis in Latine children?						
Limited access to primary care	3.35 (1.10)	0	1 (8)	6 (55)	3 (27)	1 (9)
Limited access to autism or developmental specialists	3.78 (0.80)	0	1 (8)	3 (25)	5 (42)	3 (25)
Language differences between providers and patients/families	4.06 (1.06)	1 (8)	0	3 (25)	4 (33)	4 (33)
Cultural differences between providers and patients/families	4.00 (0.80)	0	0	5 (42)	3 (25)	4 (33)
Limited availability of reliable screening tools	3.18 (1.50)	2 (18)	4 (36)	1 (9)	3 (27)	1 (9)
Limited availability of interpreter services	3.22 (1.40)	2 (17)	2 (17)	4 (33)	2 (17)	2 (17)
Limited parent trust in or willingness to communicate with the provider	3.28 (0.90)	0	2 (17)	5 (42)	4 (33)	1 (8)
Parent beliefs about normal child development and behavior	3.56 (1.00)	0	2 (17)	4 (33)	2 (17)	4 (33)
Parent understanding of the importance of early autism diagnosis and treatment	3.72 (0.80)	0	0	5 (42)	5 (42)	2 (17)
Logistical issues, such as location of services or clinic hours	3.71 (1.00)	0	1 (9)	4 (36)	3 (27)	3 (27)

#### Identifying barriers to autism assessment and diagnosis

Clinicians and care coordinators endorsed the highest impact barriers to timely autism diagnosis as being language (M = 4.06; SD = 1.06) and cultural differences between clinicians and families (*M* = 4.00; *SD* = 0.80). The barriers with the least reported impact were limited availability of reliable screening tools (*M* = 3.18; *SD* = 1.50) and limited availability of interpreter services (*M* = 3.22; *SD* = 1.40). When questioned about where in the diagnostic pipeline are Latine children more likely to experience a delay or interruption in care, clinicians and care coordinators overwhelmingly endorsed accessing autism diagnostic services (e.g., developmental pediatrician, psychologist, specialty autism clinic; *n* = 10, 56%). The second most common stage at which barriers occur is the initial screening stage whereby physician observations and caregiver concerns are first documented (*n* = 5, 28%). Clinicians ranked the top three strategies to improve early diagnosis in Latine children as increased availability of autism or developmental specialists (*n* = 9; 75%), improved parent knowledge and awareness of autism symptoms (*n* = 6; 50%), and improved referral to specialists tracking and monitoring system (*n* = 6, 50%).

#### Language interpretation and services

Around half of clinicians (*n* = 7, 58%) rated their Spanish language fluency as “good” or “excellent,” with nearly all clinicians communicating with Spanish speaking families either directly (*n* = 6, 50%) or through an in-person interpreter (*n* = 5, 42%). Most clinicians were “very” satisfied with this approach (*n* = 9, 75%). Most of the care coordinators (*n* = 4, 67%) rated their Spanish language fluency as “good” or “excellent.” Communication with families occurred either directly in Spanish (*n* = 3, 50%), with an in-person interpreter (*n* = 1, 17%), or using a telephone interpreter (*n* = 2, 33%). Most care coordinators were “very” satisfied with this approach (*n* = 4, 67%).

### Clinician and care coordinator qualitative findings

Four major themes emerged from the interviews: (1) unique patient demographics; (2) barriers to care and diagnostic evaluation; (3) challenges providing care in Spanish; (4) cultural factors that impact shared understanding. Selected quotes are provided below and in **Table 3**.

All clinicians and care coordinators reported providing care to Spanish-speaking patients, most notably individuals from Mexico (*n* = 16), Central America (*n* = 13; e.g., Guatemala, El Salvador, Honduras, and Nicaragua), and Caribbean Islands (*n* = 10; Puerto Rico and Dominican Republic); patients were primarily economically disadvantaged (*n* = 18) and a mixture of recent immigrants (*n* = 10; i.e., child not born in the United States) and established families (*n* = 13; i.e., child born in the United States). Clinicians and care coordinators reported several barriers to diagnostic evaluation and subsequent care for Spanish-speaking patient populations. Language was the most commonly endorsed barrier (*n* = 14) and the nuances involved in language such as the variety of dialects in Spanish (*n* = 8) and differences in expectations summarized by this quote, “I see that physicians have different expectations for kids who speak more than one language like lower expectations for their development and so my Hispanic families are more likely to be told to wait and see” (PID#21). Additional barriers included long waitlists (*n* = 8), insurance (*n* = 10) and family priorities based on cultural differences (*n* = 9), highlighted by this quote, “another thing I sometimes struggle with [patients] is they think they can fix it as a family or like this is the family unit. They can come together and that will be enough. But I also want my families to take advantage of every opportunity given to them. I love the family unit approach that they have, but I know for a lot of these kids it’s unfortunately not going to be enough. And that’s more the nature of the disease as opposed to any lack on their part of the family” (PID#25).

Regarding challenges providing care in Spanish, three subthemes emerged including (a) challenges with interpreters, (b) personal comfort or ability to provide care in Spanish, and (c) institutional and personal adaptations that impact care provision. Regarding interpreters, nearly all clinicians and care coordinators (*n* = 14) reported concerns with interpreters that impact the validity of the evaluation and may lead to communication difficulties, such as different dialects of Spanish used between patient and interpreter, wide range of accuracy in interpreting exactly what the patient is saying, and privacy concerns or depersonalization of the interaction between the provider and the family. For example one clinician reported, “I think there’s a delicacy in giving diagnoses and I think when you use an interpreter, it pulls out some of that personal relationship and makes it more of just like hearing it from an unattached [person],… and I found since I understand Spanish, that the interpreters will not always word things in what I was getting at … they’re saying things slightly different than what I said, but the meaning changes enough that I feel like it’s not going to come across the same way” (PID#21). Personal comfort varied based on the clinician or care coordinator’s level of bilingualism. Those who were fluent in Spanish or had a trusted interpreter or bilingual staff member (e.g., bilingual nurse) reported fewer challenges and perceived richer relationships with their patients (*n* = 12). Finally, all clinicians and care coordinators (*n* = 18) reported on a variety of personal and institutional adaptations that they implemented for care interactions. As the interviews unfolded a split occurred where clinicians who worked for institutions that prioritized system-wide adaptations (*n* = 7; e.g., bilingual staff at multiple levels; translated materials; in-person interpreters; longer visits; and a multilingual resource support center) that improved patient care delivery reported reduced challenges in providing care to Spanish-speaking patients. Alternatively, clinicians who had limited access to institutional adaptations and relied on limited support or informal personal adaptations (*n* = 5; e.g., personal approach in clinical manner; attending cultural humility trainings) reported an increase in challenges providing adequate care and support to Spanish-speaking patients.

Finally, clinicians and care coordinators reported on cultural factors that impact shared understanding. Five clinicians shared that community stigma and differing family views affect shared understanding and decision making. For example, one clinician shared “the potential for greater stigma among, just acceptance of autism spectrum disorder generally could be a challenge to some of those next steps in the needs of the family … and acceptance from the different stakeholders in the family could be a potential barrier to them” (PID#28). Care coordinators and clinicians also reflected on different cultural values, expectations, and family priorities that impact shared decision-making (*n* = 12), such as cultural differences in developmental expectations (e.g., later language development is more accepted or normed by family), and child rearing norms or family dynamics highlighted by a Latine care coordinator: “American culture is that when the child [is an] adult, they need to move out of the house. But the Hispanic culture they can stay in the house till they’re older, they don’t have to leave. And, especially if you have a child with a disability, you don’t want that child to move out of the house just because they are an adult. So sometimes they feel pushed to the train, get them to move out of the house, but that’s not what they want, that’s not part of their beliefs” (PID#36). Finally, nearly all care coordinators (*n* = 5) and all clinicians (*n* = 12) reported that Spanish-speaking Latine patients demonstrate limited and often incorrect knowledge about autism that creates challenging conversations between clinicians and patients. Moreover, diagnostic conversations may be also complicated by negative reactions to the diagnosis (*n* = 3) or gender differences in diagnosis buy-in based on traditional patriarchal hierarchies that lead to hesitancies (*n* = 7) in following through with service provision particularly by fathers (*n* = 8; e.g., “if I’m gonna get more resistance from a parent, it’s typically the father, and the mother will be more willing to try and buy-in because the mom is the one who’s home with the kids” PID#25).

**Figure 1:**
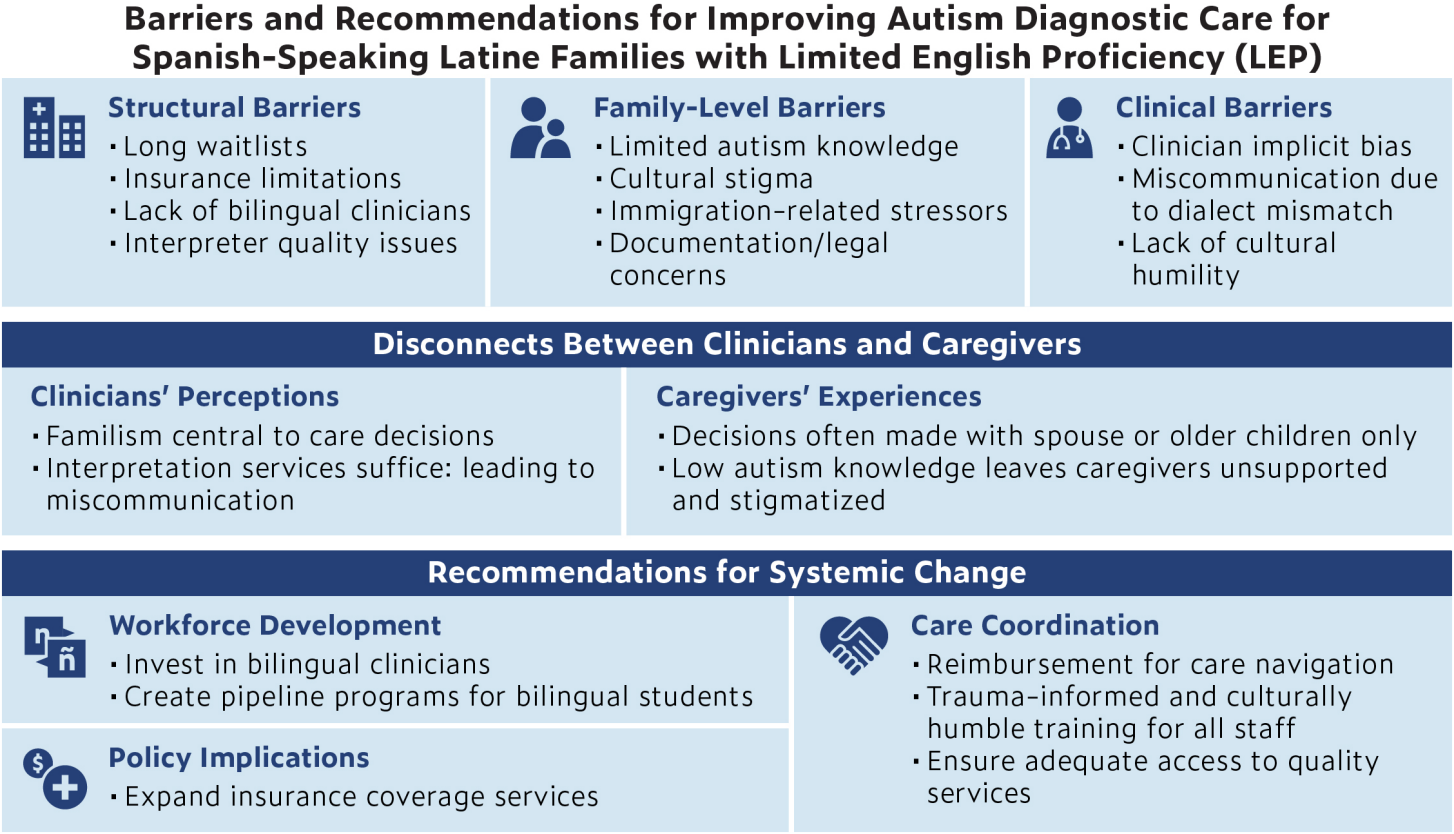
Highlights structural, family, and clinical barriers experienced by Latine families of LEP, disconnect between clinician perspectives and caregiver experiences, and recommendations for systemic change and and barrier mitigation.

See **Figure 1** for a graphic synthesis of barriers identified by participants, the highlighted disconnect between clinicians and caregivers, and recommendations for system level change to reduce barriers for Spanish-speaking LEP Latine caregivers accessing early autism diagnostic services.

## Discussion

Elucidating and understanding the barriers that delay access to autism diagnostic care is essential to building equitable models of early autism diagnosis and subsequent care for Spanish-speaking Latine children. To date, the bulk of autism diagnostic adaptation work has occurred in non-English speaking countries; cited limitations to this approach include reliance on translation alone (as opposed to cultural adaptation), lack of methodological transparency (affecting reproducibility), and insufficient attention towards cultural values and customs (23). Thus, the current study utilized a participatory approach with Spanish-speaking Latine caregivers of children with autism and clinicians that care of them to characterize barriers in acquiring an autism diagnosis and related support services for children from Spanish-speaking LEP families in the United States. A further goal of this work was to inform the development of recommendations for scalable strategies to adapt models of care that reduce barriers, improve access, and meet the cultural needs of LEP Spanish-speaking Latine families. Below, the three key domains of findings from this research are discussed.

### Limited access to knowledge and education

Caregivers noticed developmental differences at a mean age of 1.75 years and raised concerns with clinicians at approximately 2.50 years; yet formal autism diagnosis occurred later at 4.20 years. This lag between developmental concern and diagnosis mirrors previous research (6) and underscores a critical gap in knowledge and guidance. Despite early recognition, most caregivers indicated uncertainty about where to seek assistance, and fewer than half received useful information from healthcare providers. These findings align with literature documenting language and cultural barriers, stigma, and lack of culturally competent care among Latine families of autistic children (7). Clinician data revealed high self-rated autism knowledge, yet Spanish-speaking Latine parents were perceived as having the least knowledge, reinforcing implicit biases previously reported in the literature (13). This disconnect highlights the urgent need for culturally informed training that moves beyond surface level cultural generalizations to address the nuanced lived experiences of Latine families (e.g., reflected in the broader shift in emphasis on cultural humility, not just competence; 31) and targeted education for caregivers. Moreover, improving access to culturally and linguistically tailored educational materials within existing touchpoints for families, such as pediatric offices, early intervention processes, and developmental preschool evaluations. Health systems, Universities, and community-based organizations can partner to co-develop these resources with Latine caregivers to ensure cultural relevance and accessibility. Finally, embedding patient navigators or community health workers such as *promotoras* (32) within clinics may provide ongoing guidance and education for LEP families.

### Challenges and barriers in accessing diagnostic services

Language and cultural factors emerged as salient barriers to timely autism diagnosis. Several caregivers reported that clinicians attributed developmental delays to speaking Spanish at home, and one quarter delayed service engagement due to documentation concerns. Immigration-related stressors, residency status, and citizenship documentation challenges further discourage care-seeking as reported in Zuckerman et al. (19). Qualitative findings revealed additional complexities and multifaced barriers. For example, several clinicians reported challenges having conversations with caregivers and barriers such as cultural beliefs about developmental norms and the family unit. While clinicians may be alluding to *familismo* (33, 34), the concept describing the importance of family in the Latine culture, caregiver narratives revealed a more nuanced and emotionally charged reality. For example, Latine caregivers of autistic children often reported feeling isolated, unsupported by their spouses, and stigmatized by extended family, especially those residing in their country of origin. These experiences were compounded by immigration-related stressors, limited social support, and cultural stigma surrounding autism, all of which contributed to caregiver distress and increased risk for mental health challenges such as depression, anxiety, and marital strife. These findings align with prior research documenting elevated stress levels among caregivers of autistic children (35), particularly in minoritized and immigrant populations (34, 36). Moreover, communication barriers, including limited Spanish proficiency among clinicians, reliance on interpreters of variable quality, and dialect mismatches led to miscommunication and reduced caregiver trust. These breakdowns not only hindered accurate information exchange but also risked reinforcing implicit biases and microaggressions, consistent with prior research on interpreter-related challenges (37). Thus, it is crucial to invest in accessible mental health support groups and programs for LEP Latine caregivers to mitigate extenuating immigration-stressors and increase social support. This could be another avenue to incorporate patient navigators or *promotoras* to identify social and immigration-related stressors and connect families to legal and social support resources through community organizations that support immigrants and refugees, medical-legal partnerships, or integrated behavioral health models.

### Opportunities for health system change

While findings do not suggest a need for major procedural overhauls in diagnostic care models for Latine children and their families, they underscore the urgent need for systemic and structural changes to improve equity in diagnostic care. Recommended strategies include: (1) investment in bilingual clinicians and staff at all levels of care. Bilingual clinicians and care coordinators can foster trust, reduce miscommunication, and improve diagnostic accuracy. In addition, in-house interpreters (rather than outsourcing to a company) for highly requested languages may also reduce variability in interpretation quality; (2) reimbursement models that support coordination of care, including care navigation and family support services critical for LEP families navigating novel and complex systems; (3) training programs that emphasize cultural humility, trauma-informed care, and the psychosocial realities of immigrant families, rather than relying on cultural generalizations to guide clinical care; and (4) pipeline programs to recruit and support bilingual students from minoritized backgrounds in entering healthcare professions aiming to address long-term workforce shortages in linguistically and culturally competent care. These strategies echo recommendations in the literature (16, 17, 21) and represent actionable steps towards reducing disparities and improving timely autism diagnosis for Spanish-speaking Latine families. Most importantly, these strategies are most likely to be effective when implemented in combination, as part of multilevel interventions that simultaneously address workforce capacity, institutional practices, and structural barriers to care.

## Limitations

Several limitations should be considered when interpreting study findings. The present study did not engage a non-Latine control group in order to ascertain whether the reported challenges are specific to the Latine community or more broadly reported across autistic children in the US. Moreover, sample sizes for the three groups (clinicians, caregivers, care coordinators) were relatively small, which may have limited statistical power and generalizability of findings. Participants were largely drawn from the Midwest, specifically large cities of higher urbanicity, which may not reflect experiences in other regions of the state and the country (e.g., rural areas) or those who are members and consumers of different healthcare systems. Additionally, data rely on caregiver, clinician, and care coordinator self-report, which may be subject to recall bias and social desirability effects, particularly in the focus group findings (38). Also, the study employed a cross-sectional design, capturing perceptions at a single time point, preventing conclusions about changes over time or causal relationships. As such, our findings should be interpreted with these limitations in mind and future research should include larger comparative studies of caregiver, clinician, and care coordinator experiences and reflections across multiple time points over the long term autism diagnostic journey. Finally, while a focus on predominately Spanish-speaking Latine participants is a strength of the current study, future research may seek to explore perceptions across a broader and diverse sample of languages and cultural groups, to capture different experiences of similar challenges.

## Conclusion

This study highlights three key challenges impacting delays in autism diagnosis among Spanish-speaking LEP Latine families. Limited access to knowledge and education about autism plays a key role in furthering long-standing community stigma, even in situations where Latine families are reporting early caregiver developmental concerns. Systemic barriers, such as clinician implicit biases, language, and cultural differences between clinicians and caregivers, create challenges in accessing diagnostic services. Finally, there continue to be missed opportunities for health system changes to support access for early autism diagnoses. These findings have important implications for policy and practice. Addressing language access and cultural responsiveness must go beyond translation services (e.g., interpreters) to include structural investments in workforce development, institutional adaptation, and equitable reimbursement for coordinated care. Only through such comprehensive multi-level efforts can we ensure all children, regardless of language or background, receive timely, high-quality autism diagnostic care.

## Data Availability

The datasets presented in this article are not readily available because the dataset includes a small number of participants who discuss personal and potentially identifiable information. Requests to access the datasets should be directed to martannm@iu.edu.
